# Effectiveness of Motivational Interviews for Alleviating Pre- and Postoperative Anxiety and Postoperative Pain Among Patients Undergoing Surgery: A Systematic Review and Meta-Analysis

**DOI:** 10.3390/medicina62061182

**Published:** 2026-06-18

**Authors:** Celia Villalba-Aguilar, José Alberto Laredo-Aguilera, Lucía Villalba-Aguilar, Laura Pilar de Paz-Montón, Víctor Serrano-Fernández, Juan Manuel Carmona-Torres

**Affiliations:** 1 Solimat Hospital, 45004 Toledo, Spain; celia.villalba@alu.uclm.es; 2 Faculty of Physiotherapy and Nursing of Toledo, 45071 Toledo, Spain; josealberto.laredo@uclm.es (J.A.L.-A.); laurapilar.paz@uclm.es (L.P.d.P.-M.); 3 Multidisciplinary Research Group in Care (IMCU), University of Castilla-La Mancha, 45071 Toledo, Spain; 4 Health Research Institute of Castilla-La Mancha (IDISCAM), 45004 Toledo, Spain; 5 Faculty of Health Sciences, Rey Juan Carlos University, 28922 Alcorcón, Spain; luciavillalbaaguilar12@gmail.com; 6 Department of Nursing, Faculty of Medicine, Autonomous University of Madrid, 28029 Madrid, Spain; victor.serrano@uclm.es

**Keywords:** preoperative anxiety, nursing, postoperative anxiety, postoperative pain

## Abstract

*Background and Objectives*: Anxiety can influence the intensity of postoperative pain, anesthesia and the need for analgesia. Likewise, proper anxiety management can reduce hospital stays. Therefore, it is important to review the actions of nursing professionals and estimate the effect size of nursing interventions to reduce anxiety in pre- and postsurgical processes. Therefore, the objective of this study was to analyze the effectiveness of preoperative motivational interviews by nursing to reduce pre- and postoperative anxiety and postoperative pain after surgery. *Materials and Methods*: A systematic review was carried out according to the PRISMA guidelines, with a record in PROSPERO using DeCS and Boolean operators OR and AND to increase the specificity of the search. In the EBSCOhost, Pubmed, Cochrane Library and Scopus databases, 104 studies were obtained. Patients ≥ 18 years of age with scheduled surgeries, communication skills, clinical trials or quasi-experimental studies were included. The risk of bias 2 (RoB2) tool was used to assess the risk of bias in the studies. A random effects meta-analysis was performed to analyze pre- and postoperative anxiety and postoperative pain. *Results*: A total of 6 studies were included (4 clinical trials and 2 quasi-experimental studies). All the studies analyzed preoperative anxiety, which was significantly lower (SMD = −1.53; 95% CI: −4.01 to −0.95; I^2^ = 40%). Postoperative pain (SMD = −0.74; 95% CI: −0.94 to −0.54; I^2^ = 35%) and postoperative anxiety (SMD = −0.48; 95% CI: −0.78 to −0.19; I^2^ = 0%) also decreased. *Conclusions*: Motivational interviews by nurses may help provide clearer information and emotional support and allow patients to express doubts, reducing their fear of anesthesia, pain and the recovery process. Emotional control improved adaptation to the surgical environment, reducing postoperative pain. With respect to implications for clinical practice, it is necessary to use the same scales to measure anxiety to better compare the studies.

## 1. Introduction

Anxiety is an emotional response to stimuli perceived as threatening [1,2]. These responses not only affect the psychological and emotional spheres but also have physiological and functional consequences [1,2]. According to some authors, anxiety in normal conditions improves adaptation to the external environment to avoid or face danger. However, when the body reacts in a maladaptive way and when the reaction is exaggerated, anxiety is considered a mental disorder [1].

On the other hand, anxiety can be viewed in two ways: as a trait or as a state. Trait anxiety is part of a person’s personality; it is their habitual tendency to feel anxious. State anxiety, however, is a temporary reaction to a specific moment, such as the physical response a patient feels right before surgery. This type of response typically occurs in patients who have undergone surgery [1,3]. Preoperative anxiety, characterized by diffuse fear, can range from restlessness to panic [4]. It is an unpleasant emotional state experienced by those who are waiting to undergo surgical intervention, which begins when the need for surgery is communicated to the subject, reaching its peak immediately before entering the operating room [5], and can influence postoperative pain intensity, anesthetic requirements, and the need for analgesia. In certain types of surgery, anxiety can even increase postoperative morbidity and mortality [6]. The main contributing factors include a lack of clinical information regarding both surgery and anesthesia, concerns over potential complications and side effects, the loss of autonomy (dependence on others), and the fear of mortality [7].

According to the most recent data from the Spanish Ministry of Health, in 2024, approximately 3,708,500 surgical interventions were performed in the hospitals of the National Health System (NHS) without accounting for the interventions derived from private health care [7,8]. The World Health Organization (WHO) noted that each year, more than 4 million surgeries are performed on patients, and it is estimated that between 50% and 75% of them experience some level of anxiety before the operation. For this reason, anxiety is considered a public health problem because it affects 10% of the global population [2].

Since the emergence of the nursing profession, the work of nurses has been linked to individualized care, empathy, closeness and patient-centered care [7]. In her work “Notes on Nursing: What it is and what it is not”, Florence Nightingale stated that nursing should not be limited only to administering medications and applying superficial treatments. However, regarding the care provided by nursing professionals in the surgical area, perioperative nursing care is often perceived as a predominantly technical task, with the emotional dimension of patients being ignored or overlooked [7,8]. However, this is not the case since the nursing staff is key in the perioperative process. From the preoperative period, they perform a presurgical interview, where they must verify and identify the risk factors for surgery, evaluate the needs of the patients, manage their expectations, and understand their vulnerabilities until the postoperative period, when patients are monitored and vital signs are assessed [1,2].

Several studies have shown that preoperative nursing visits have positive effects on anxiety management among surgical patients. However, there is some controversy about the treatment of patients’ anxiety and the information that should be provided [7]. Rojas et al. (2013) [4] reported that individuals experience many fears, but it is necessary to always listen to patients, apply empathy, identify sources of concern, and provide adequate information to help mitigate their anguish. Several authors have reported that anxiety management can reduce hospital stay and improve postsurgical pain. Other studies suggest that excessive information provided to highly anxious patients may increase anxiety levels [7]. However, it is a nursing task to carry out actions to facilitate communication, clarify doubts, correct misconceptions, and reduce fear of the unknown, given that numerous studies have demonstrated a high prevalence of perioperative anxiety. Before they reach the operating room, psychological support becomes the fundamental pillar for them to come calmly. Furthermore, NIC interventions are aligned with recommendations from the Ministry of Health, and the NOC results indicate that a “decrease in anxiety” will guarantee the well-being of the patient in the surgical block [3,4,7].

Considering the importance of reducing anxiety in the pre- and postoperative periods, it is important to review the actions of nursing professionals and estimate the effect size of nursing interventions to reduce anxiety in these situations [1].

Patients undergoing surgery frequently interpret the perioperative experience as a threat characterized by uncertainty, loss of control, and fear of adverse outcomes [9]. Traditional preoperative education in hospitals is often limited to the delivery of standardized biomedical information and procedural instructions, adopting a predominantly one-directional and task-oriented communication style [10]. Although this approach may improve procedural understanding, it does not always address the patient’s emotional needs, cognitive appraisal of surgical danger, or individual coping resources. In contrast, motivational interviewing (MI) represents a patient-centered communication strategy that promotes active participation, emotional expression, and shared decision-making. Through its core principles of partnership, acceptance, compassion, and evocation, MI may help patients reinterpret the surgical experience as more manageable and controllable, thereby reducing state anxiety and attenuating stress-related physiological responses associated with postoperative pain perception [9].

MI is a collaborative communication framework focused on the ‘language of change.’ Rather than imposing external pressure, it aims to strengthen an individual’s intrinsic motivation and commitment by exploring personal concerns and encouraging adaptive coping strategies within a supportive and empathetic environment. Unlike traditional preoperative education, which is frequently directive and information centered, MI emphasizes the therapeutic relationship and the patient’s self-efficacy. By fostering trust, emotional support, and perceived control, MI may contribute to decreasing preoperative anxiety and modulating postoperative pain experiences.

In recent years, several meta-analyses have been carried out to analyze how these interventions reduce preoperative anxiety. For example, many researchers have studied different interventions to reduce preoperative anxiety, such as music therapy, art therapy, clown therapy, therapeutic play and hand massage [11], among others, with positive results. However, to our knowledge, no previous studies have performed meta-analyses on the efficacy of interventions based on motivational interviews by nurses for pre- and postoperative anxiety and postoperative pain.

Therefore, the objective of this study was to determine the effectiveness of pre- and postoperative motivational interviews by nursing professionals with patients undergoing surgery in reducing presurgical anxiety and postoperative pain.

## 2. Methodology

### 2.1. Design

This systematic review was conducted in accordance with the Preferred Reporting Items for Systematic Reviews and Meta-Analyses (PRISMA) statement (Supplementary Material S1) [12]. In addition, this systematic review was registered in the PROSPERO (International Prospective Register of Systematic Reviews) under registration number CRD420251062404 [13].

### 2.2. Information Sources

A literature search was conducted in May 2025 in the following databases: EBSCOHOST, PubMed, the Cochrane Library and Scopus.

The literature search strategy mentioned in Table 1 was used.

### 2.3. Search Strategy

The formulation of the research question was developed on the basis of the PICO question format as follows: do motivational interviews conducted by nurses (I) reduce pre- and postoperative anxiety and postoperative pain (O) in patients undergoing surgery (P) compared with usual care (C)?

### 2.4. Selection Criteria

To ensure the rigor and internal validity of this meta-analysis, specific inclusion and exclusion criteria were established. Studies were eligible for inclusion if they were clinical trials or quasi-experimental studies involving an adult population (≥18 years) undergoing scheduled (elective) surgeries. Owing to the nature of the intervention, the included studies were required to focus on patients with preserved communication skills to ensure active engagement in the therapeutic dialog. No language restrictions were applied to the initial search, although the final synthesis focused on studies published in English, Spanish, or Portuguese.

The decision to include only clinical trials and quasi-experimental studies in this systematic review is based on the highest quality of scientific evidence possible.

Conversely, several exclusion criteria were applied to minimize confounding variables. Patients with a psychiatric history or cognitive impairment were excluded because motivational interviewing relies on the patient’s capacity for cognitive reflection and “change talk,” which may be compromised in these populations. Similarly, patients with severe communication barriers (such as profound hearing loss or language barriers) were excluded to ensure the technical fidelity of the intervention. To maintain a more homogeneous psychological profile, this study excluded emergency surgeries, as the acute psychological state in life-threatening situations differs fundamentally from that of elective procedures. For the same reason, high-complexity cases (specifically organ transplantations, oncology patients, and pregnant women) were omitted, as these populations experience unique physiological and psychological stressors that could distort the assessment of generalized surgical anxiety.

With respect to the surgical setting, trials involving outpatient surgeries or those with a postassessment period of less than 24 h were excluded to allow for a standardized evaluation of postoperative pain and analgesic requirements. Finally, to ensure clinical relevance and alignment with modern ERAS (enhanced recovery after surgery) protocols, the review was limited to studies published within the last 10 years and excluded studies with descriptive or observational designs and animal-based research.

### 2.5. Selection of Studies and Data Extraction

The literature search and study selection were independently conducted collaboratively by two independent reviewers (XXX and YYYY) across the specified databases. Following the removal of duplicates, studies were screened on the basis of title and abstract. A full-text review was subsequently performed to ensure compliance with the predefined inclusion and exclusion criteria. Disagreements between reviewers were resolved through consultation with a third reviewer (ZZZZ).

### 2.6. Assessment of Risk of Bias

Methodological quality was evaluated following the methods of the Cochrane Collaboration. Specifically, the Rob 2 tool (RoB2) [14] was employed to assess randomized clinical trials and [15,16] and quasi-experimental studies [5,17], whereas the ROBINS-I tool was used in nonrandomized studies of interventions [18].

Both frameworks utilize a domain-based structure to evaluate study design, execution, and reporting. RoB 2 evaluates six key dimensions (ranging from the randomization process to outcome reporting) using a standardized algorithm that categorizes risk as ‘low,’ ‘high,’ or ‘some concerns’ (uncertain). Similarly, the ROBINS-I examines seven distinct domains, including confounding factors, participant selection, and intervention classification, to provide a comprehensive bias profile. This tool classifies the risk of bias into five categories: “low”, “moderate”, “serious”, “critical” and “no information” [18].

### 2.7. Data Extraction

Data extraction was performed independently by researchers XXX and YYYY. For each included study, the following variables were recorded: lead author, publication year, and country of origin; study design and sample size; eligibility criteria; and details of the intervention and comparator. The primary outcomes extracted were preoperative anxiety, postoperative anxiety, and pain levels.

### 2.8. Statistical Analysis

A qualitative synthesis was carried out for each of the studies included in this systematic review. The analyzed outcomes included preoperative anxiety, pain and postoperative anxiety.

A random-effects meta-analysis was performed because of the expected clinical and methodological heterogeneity among the included studies. Differences were identified in surgical specialties, intervention duration and structure, follow-up periods, anxiety assessment instruments, and study designs, which justified the assumption that the true intervention effect could vary across studies [5,17,19,20,21,22]. Because the included studies used different instruments to measure anxiety and pain outcomes, standardized mean differences (SMDs) with their corresponding 95% confidence intervals (CIs) were calculated. The results are presented as forest plots, with statistical significance established at *p* < 0.05.

Statistical heterogeneity among studies was assessed using the I^2^ statistic and classified as low (I^2^ ≤ 25%), moderate (I^2^ = 26–50%), or high (I^2^ ≥ 51%). A leave-one-out sensitivity analysis was conducted by sequentially excluding each study and recalculating the pooled effect estimate to evaluate the influence of individual studies on the overall results and the robustness of the findings. Owing to the limited number of included studies (<10), publication bias could not be reliably assessed using funnel plots.

All the statistical analyses and data syntheses were conducted using Review Manager software (RevMan version 5.4; Cochrane Collaboration).

## 3. Results

### 3.1. Characteristics of the Included Studies

**Figure 1:**
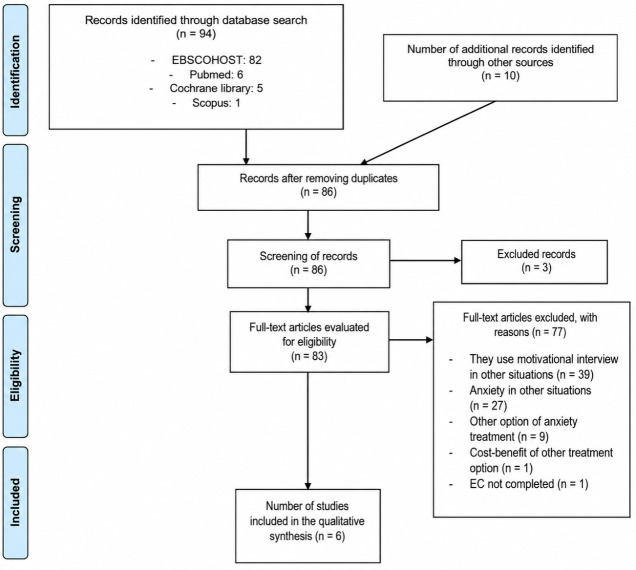
PRISMA flow chart.

A total of 104 articles were identified. After duplicate articles were eliminated, a total of 86 articles remained. After articles that did not meet the inclusion criteria were eliminated, four clinical trials were selected [19,20,21,22,23,24,25,26] and two quasi-experimental studies were selected [5,17]. The diagram used in the selection of articles for the systematic review is shown in Figure 1.

A total of 734 patients were included, with MI applied to 334 patients. All included studies involved participants aged ≥ 18 years.

In all the studies, the ethical standards of the committees of the hospitals where they were carried out were followed, in addition to the ethical guidelines for medical and health research in humans through the Declaration of Helsinki.

Table 2 describes the main characteristics of the 6 studies included in the review, revealing that they have been carried out in different countries (3 in Spain, 1 in Switzerland, 1 in Arabia and another in Colombia). In turn, these studies demonstrate the different durations of the intervention and the follow-up periods, which contribute to the variability in different populations and settings. The number of participants in each study ranged from 56 to 292. The age range of the participants was between 18 and 87 years.

### 3.2. Types of Interviews Conducted

Table 3 describes the interviews conducted in each of the studies included in this review. In general, they were interviewed with more specific information about the interventions, which were carried out by the nursing staff.

Notably, the interviews ranged from 10 min [20] to 40 min [22]. Similarly, 2 of the studies conducted interviews 20 days before the day of surgery [17,22]. Only one study indicated that relatives and patients were informed [5].

### 3.3. Risk of Bias

**Figure 2:**
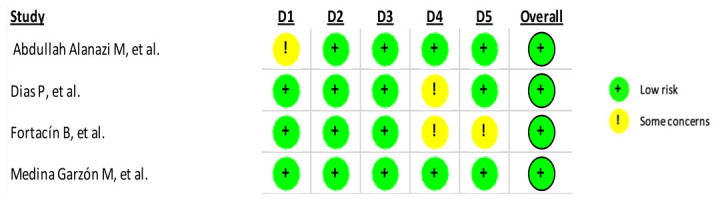
Assessment table of biases in the literature of the included clinical trials [19,20,21,22].

**Figure 3:**
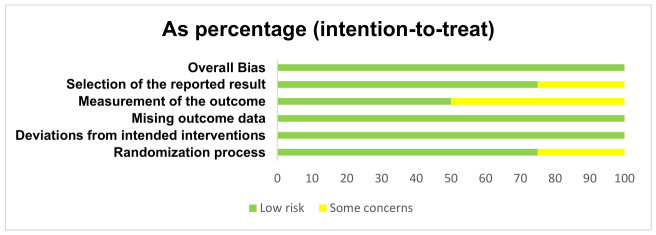
Overall risk of bias in clinical trials.

The risk of bias assessment was evaluated with the Rob2 tool, as shown in Figure 2 and Figure 3.

The evaluated studies have a design with appropriate randomization, correct handling of the data and adequate statistical analysis, which strengthens the internal validity of their results. Therefore, although randomization and analysis are generally adequate in all studies, the absence of blinding and certain methodological aspects, in some cases, represent a moderate risk of bias, especially in the measurement of subjective outcomes, such as in the measurement of postoperative anxiety and patient satisfaction. However, no study was excluded from the review because, despite some methodological concerns, all the studies were considered to provide relevant information for the objectives of the review.

**Figure 4:**
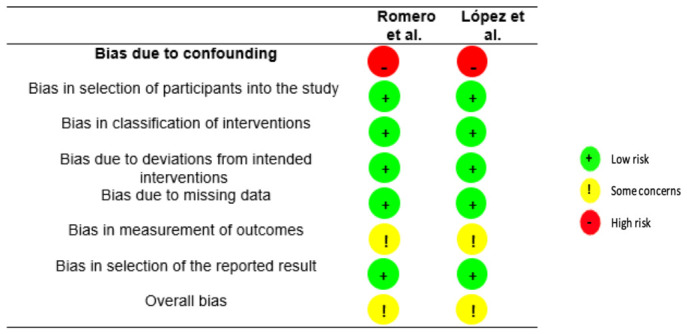
Assessment of biases in the literature of the included quasi-experimental studies [5,17].

The ROBINS-I tool was used to assess the risk of bias in nonrandomized studies of interventions [5,17]. These results are shown in Figure 4.

No study was randomized, and statistical adjustment was not reported to control for variables that may influence the results. With respect to the selection and classification of the participants, the groups were clearly defined and assigned before the results were measured, and validated scales were used to measure the level of anxiety [5,17] and pain [17]; no major data losses were reported. However, the lack of blinding of both participants and assessors may have influenced the responses, generating a moderate risk of bias. In turn, no significant risks of bias were identified in the selection of the reported outcome.

Therefore, the two studies had a moderate risk of bias, mainly because of the possibility of uncontrolled confounding, nonrandomization and unblinded measurement of the results.

The methodological quality was independently reviewed by the XXX and YYYY investigators. The reliability between the authors was high, and any disagreements were discussed with ZZZZ until an agreement was reached.

### 3.4. Preoperative Anxiety

All 6 articles included in the review addressed preoperative anxiety. However, some data from 2 of the articles are needed. Notably, according to Romero et al. [5], 60% of the CG patients according to the APAIS scale had preoperative anxiety, whereas 46% of the IG patients did. Similarly, in this study, females reported more anxiety than males did. Fortacín et al. [21] assessed preoperative anxiety using the STAI scale, in which the average CG score was 15.83 points and the mean IG score was 3.03 points. López et al. [17] evaluated preoperative anxiety with the Hamilton Scale and reported 18 points on average for the CG and 10 points on average for the IG, and the difference was statistically significant (*p* < 0.0001). Notably, in the study by Dias et al. [20], 17% of the IG and 13% of the CG were given anxiolytic medication.

**Figure 5:**
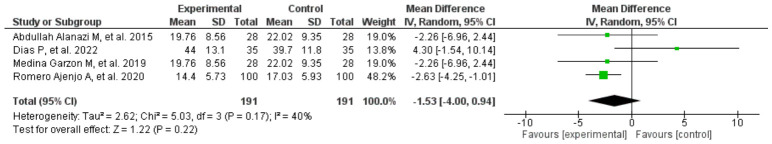
Forest plot of preoperative anxiety [5,19,20,22].

The results of the meta-analysis regarding preoperative anxiety are shown in Figure 5. Compared with regular interviews, motivational interviews by nurses decreased preoperative anxiety, with an SMD = −1.53 (95% CI between −4.00 and −0.94). A random effects model was used because the statistical heterogeneity was moderate (I^2^ = 40%).

### 3.5. Postoperative Pain

Three of the 6 articles analyzed postoperative pain [19,20,22]. Notably, in this study, López P et al. [17] did not provide data on the mean and standard deviation (SD) and revealed that 21% of the CG patients required morphic chloride rescues, whereas 11% of the IG patients did.

**Figure 6:**
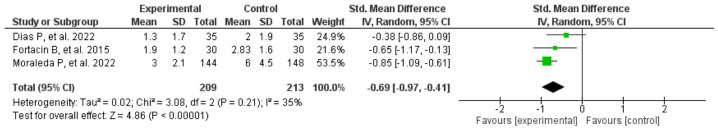
Forest plot for postoperative pain [17,20,21].

The results of the meta-analysis regarding postoperative pain are shown in Figure 6. Compared with the usual interviews, the motivational interviews by nurses decreased postoperative pain, with an SMD of −0.69 (95% CI between −0.97 and −0.41).

### 3.6. Postoperative Anxiety

**Figure 7:**
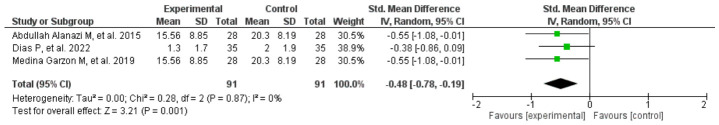
Forest plot for postoperative anxiety [19,20,22].

Regarding postoperative anxiety, 3 of the 6 articles analyzed these results [19,20,22]. The results of the meta-analysis regarding postoperative anxiety are shown in Figure 7. Compared with regular interviews, motivational interviews by nurses decreased postsurgical anxiety, with an SMD = −0.48 (95% CI between −0.78 and −0.19).

### 3.7. Sensitivity Analysis

The results of the leave-one-out sensitivity analysis are presented in Table 4. A leave-one-out sensitivity analysis was performed to assess the robustness of the pooled estimates. With respect to preoperative anxiety, the exclusion of individual studies resulted in some variation in the magnitude of the pooled effect and heterogeneity. The greatest change was observed after removal by Romero et al. [5], who reduced the effect size and rendered the pooled estimate nonsignificant. Nevertheless, the direction of the effect consistently favored motivational interviewing. With respect to postoperative pain and postoperative anxiety, the sequential exclusion of individual studies did not substantially modify the pooled effect estimates or their statistical significance. Heterogeneity remained low or absent in most analyses. Overall, the findings suggest that the beneficial effects of motivational interviewing on perioperative outcomes were generally robust, although the results for preoperative anxiety appeared to be more sensitive to the influence of individual studies.

## 4. Discussion

The observed results show that MI by nurse professionals reduces pre- and postoperative anxiety. However, different types of scales have been used to measure both pre- and postsurgical anxiety. The one that was used most [5,19,22] was the Amsterdam Preoperative Anxiety and Information Scale (APAIS), which assesses anxiety related to anesthesia and surgery and the need for additional information [27]. The second most commonly used scale [20,21] is the State-Trait Anxiety Inventory (STAI), which is a psychological scale to measure anxiety in adults and assesses two different components of anxiety: state anxiety, which measures transitory anxiety, that is, how a person feels at a specific time (for example, before or after surgery), and trait anxiety, which assesses the general tendency to react with anxiety to stressful situations [28]. In the study by López et al., only the Hamilton Scale was used to measure the severity of patients’ anxiety [17]. This scale consists of 14 items that evaluate psychological and somatic symptoms [29]. However, in the article of Rojas et al. [4], the level of anxiety was measured via the Beck test after an educational nursing strategy, which was used during the pre- and postoperative periods, which was consistent with our results. On the other hand, several studies involving outpatient surgery patients have reported that empathic intervention focused on patients can increase surgical recovery, wound healing and patient satisfaction and reduce preoperative anxiety [30,31,32,33].

Fortacín et al. [21] reported that patients with a low level of education had higher anxiety scores than did Kindler et al. [34] and Herrera et al. [35] did.

On the other hand, Romero et al. [5] reported that anxiety is greater among women than among men.

In a single study, information is given to the patient’s family [5]. Thus, it has been shown that the relatives of patients who are going to be operated on and who express doubts about the process are given the information correctly, offering ethical and dignified care so that their attitudes influence these patients, especially those of advanced age [21,36].

Although audiovisual materials are effective at increasing patient satisfaction, they do not reduce anxiety [37,38,39]. Therefore, for those patients who are going to undergo surgery, MI appears to be effective because patients receive clearer information and greater emotional support with questions and dialogs that have helped them reduce the fear of anesthesia, pain, and separation from family members and consider how the recovery process will occur [40]. This is also demonstrated by Howick et al. [41], who reported that empathy can reduce anxiety and pain in patients, increase their level of satisfaction and, therefore, have effects similar to those of usual pharmacological treatments. Thus, communication skills are essential for nursing professionals caring for surgical patients [19]. Kerper et al. [42] reported that motivational interviews are valuable tools for reducing preoperative anxiety. As suggested by some authors, conventional interventions do not effectively reduce anxiety because nurses must engage in empathic and collaborative patient communication. This involves providing informative and persuasive support to mitigate anxiety while enhancing the overall surgical experience and patient satisfaction [19,43,44,45].

The studies analyzed have also shown a decrease in postoperative pain [19,20,22], which is also supported by other studies in which the emotional state of the patient, both owing to a lack of knowledge and the circumstances surrounding the surgical process, affects their ability to adapt and increases pain [46]. The findings of the study by Pereira et al. [30] agree with our findings because pain improved after motivational interviews. However, Dias et al. [20] reported that opioid consumption and pain on the second day were similar in both groups, as was postoperative anxiety. This is because the intervention that was used focused on aspects related to surgery and not on the postoperative period. Therefore, another interview after the operation should also be considered. Similarly, poor pain control can negatively affect quality of life and functional recovery and increase the risk of postsurgical complications; therefore, poor pain control is associated with increased morbidity and hospital costs, increasing the risk of developing persistent chronic pain [17].

One of the main limitations of the included studies was the heterogeneity in the instruments used to measure anxiety during the pre- and postoperative periods. Various scales were used, such as the APAIS, STAI, and Hamilton scales, each with different approaches, psychometric properties, and cutoff points. This variability in measurement tools makes direct comparisons between studies difficult and may influence the interpretation of the magnitude of the observed effects. In addition, this reflects the lack of standardization in the evaluation of anxiety in surgical contexts, highlighting the need for methodological consensus to improve consistency and comparability in future research.

The sensitivity analysis confirmed the overall stability of the findings, particularly for postoperative pain and postoperative anxiety. Nevertheless, some variability was observed for preoperative anxiety, indicating that this outcome may be more susceptible to the influence of individual studies and methodological differences.

The heterogeneity observed across studies may also be explained by important clinical and methodological differences. The duration and structure of the motivational interviewing interventions varied substantially, ranging from brief 10 min perioperative interviews to multiple sessions conducted over several weeks. Furthermore, the included studies involved different surgical specialties and perioperative contexts, which may influence baseline anxiety levels, pain perception, and postoperative recovery expectations. Variability in nurse training, communication styles, and intervention timing may have contributed to differences in effectiveness among studies.

The inclusion of both randomized and quasi-experimental studies may have increased methodological heterogeneity and reduced the internal consistency of the pooled estimates. Therefore, the findings should be interpreted with caution.

Another limitation is that some studies did not randomize the subjects because the noncontamination of the CG could not be guaranteed. No cause-effect can be assured, but only a relationship can be assured [5].

However, in one of the trials, a single nurse carried out motivational interviews, guaranteeing the homogeneity of the intervention and the specific knowledge of the procedure to avoid biases [20].

As strengths, nursing training in communication skills, specifically in patient-centered empathic approaches, demonstrates that preoperative interviews can be used routinely. This intervention is presented as an effective, nonpharmacological and patient-centered tool. In turn, these effects are maintained even in different types of surgeries, including ambulatory surgeries. The importance of informing and caring for family members is also highlighted, thus improving the emotional environment of patients.

## 5. Conclusions

In conclusion, motivational and educational nurse-led interventions appear to reduce pre- and postoperative anxiety, as well as post-operative pain, among surgical patients. The findings highlight the importance of nurses’ communication skills and patient-centered approaches during the perioperative period, as patients who experience greater emotional support tend to report lower anxiety levels, improved pain control, and higher satisfaction with care.

Because MI does not require specialized equipment or costly resources, it may represent an efficient and feasible nonpharmacological intervention in surgical settings. By improving emotional regulation and coping strategies, MI could reduce the use of medications such as benzodiazepines and opioids, thereby minimizing adverse effects, dependency risks, and pharmacological burden, particularly in older adults. In addition, improved pain management may contribute to better recovery, lower postoperative complications, and reduced healthcare costs.

From a clinical management perspective, nurse-led motivational interviewing could be integrated into standard enhanced recovery after surgery (ERAS) pathways through brief structured perioperative interviews without substantially increasing the nursing workload. Since several included interventions were short and easily incorporated into routine care, this strategy may provide a practical and low-cost approach to improve perioperative emotional preparation and patient experience.

Finally, given the relevance of preoperative anxiety observed in the included studies, routine assessment of this variable should be considered in perioperative clinical practice. Future research should also explore patient satisfaction and long-term recovery outcomes. Furthermore, motivational interviewing may have potential applications in other healthcare settings, such as palliative care, chronic pain management, and emergency care, where emotional regulation and therapeutic communication are essential components of patient-centered care.

## Figures and Tables

**Table 1:** Search strategy used for each database.

Databases	Search Strategy
EBSCOhost	TI ((“Motivational Interviewing” OR “motivational approaches”) AND (“nurse” OR “nurses” OR “nursing”) AND (“nurse care” OR “nursing care” OR “nursing interventions”) AND (“anxiety” OR “surgical anxiety” OR “perioperative anxiety”) AND (“pain management” OR “pain relief” OR “pain control” OR “pain reduction”)) Filters: Clinical Trials; Last 10 years; Full text.
Pubmed	(“Motivational interviewing” [Title/Abstract]) AND (surgery [Title/Abstract] OR preoperative [Title/Abstract]) AND (anxiety [Title/Abstract] OR pain [Title/Abstract]) Filters: Clinical Trials; published in the last 10 years
Cochrane library	(“Motivational interviewing”):ti, ab, kw AND (“nurse OR nurses OR nursing) AND (“preoperative anxiety”): ti, ab, kw AND (“pain management” OR “pain control” OR “pain reduction” OR “postoperative pain”): ti, ab, kw
Scopus	(TITLE-ABS-KEY(“motivational interviewing”)) AND (TITLE-ABS-KEY(nurse OR nurses OR nursing)) AND (TITLE-ABS-KEY(preoperative OR surgical)) AND (TITLE-ABS-KEY(anxiety OR “surgical anxiety” OR “perioperative anxiety” OR pain OR “pain management” OR “pain relief” OR “pain control” OR “pain reduction”))

**Table 2:** Main characteristics of the studies included in the systematic review.

Study	Design	Duration and Country	Sample Size	Inclusion Criteria	Intervention	Comparator	Results	Risk of Bias
López et al. [17], 2022.	Quasi experimental study.	Seville. 2017–2019.	N = 292 IG (n = 148). CG (n = 144).	- Patients between 18 and 75 years old. - To be the first patient of the surgical part of the morning. - Specialties: Orthopedic and Traumatological surgery. Urological Surgery, General Surgery and Vascular Surgery.	A presurgical interview with surgical patients admitted to the hospitalization floors from Monday to Thursday from 7:30 p.m. to 8:30 p.m.	Patients who have not attended the presurgical interview for various reasons.	Decreased level of anxiety in the IG and postoperative pain. Decreased use of morphic chloride in the IG.	Low
Romero et al. [5], 2020.	Quasi experimental study.	Madrid. 2018.	N = 200 IG (n = 100) CG (n = 100).	- >18 years. - Patients scheduled for surgery in the surgical block of the Hospital Universitario del Sureste and who enter the same day of surgery from their home and go through the admission service. - Communication skills.	In addition to the usual clinical practice, it will be given an informative brochure, specially designed for research, with information on the perioperative care it will receive.	Routine clinical practice: patients are informed in the consultation of the corresponding surgical specialty as well as in the preanesthetic consultation and through a telephone call by the nursing staff the day before the intervention.	The information provided on perioperative care in the presurgical patient is related to lower levels of anxiety.	Low
Abdullah et al. [19], 2015.	Controlled RCT of a preventive type.	Arabia. 2018.	N = 56 IG (n = 28) CG (n = 28).	- Patients between 50 and 75 years old. - Patients by appointment for knee replacement surgery in the next two months and willingness to participate.	Training session on surgical preparation and procedures and MI.	Standard treatment.	Preintervention anxiety scores were similar in both groups. However, postintervention anxiety scores were lower in the IG.	Low
Dias et al. [20], 2022.	Single-center, prospective, open-label controlled RCT with balanced randomization (1:1).	Switzerland 2020.	N = 70 IG (n = 35) CG (n = 35).	- ≥18 years. - Consecutive patients undergoing major elective visceral surgery at Lausanne University Hospital lasting more than 2 h. - Hospitalization the day before surgery and sufficient command of the French language.	Additional dedicated and standardized 10 min interview specifically trained by nurse in the reception facility following a patient centered approach.	Routine care and procedures: Welcome interview with the anesthesia nurse based on a safety checklist, with technical and procedural aspects.	A preoperative dialog with a patient-centered approach helped reduce preoperative anxiety in patients undergoing major visceral surgery.	Low
Fortacín et al. [21], 2015.	EC controlled.	Catalonia. 2012.	N = 60 IG (n = 30) CG (n = 30).	- ≥18 years. - Scheduled admission to the hospitalization unit of the Orthopedic and Traumatological Surgery Service of the Sant Pau i Santa Tecla Hospital in Tarragona, to be operated on for total knee prothesis, total hip replacement and lumbar arthrodesis.	The afternoon before surgery, the nurse from the hospitalization unit gave standardized information for both groups. Additionally, the IG received specific information from the preoperative visit.	Standard information.	The IG shows a statistically significant decrease in the level of anxiety in the preoperative period, in postoperative pain and an increase in well-being.	Low
Medina et al. [22], 2019.	RCT controlled preventive type.	Colombia. 2018.	N = 56 IG (n = 28) CG (n = 28).	- Patients between 50 and 75 years scheduled for knee replacement with less than 2 months prior to the procedure and their acceptance to participate in the study.	Usual treatment together with a motivational interview.	Usual treatment.	The mean preoperative anxiety score was the same in the preintervention evaluation in both groups, while the postintervention score was lower in the CG.	Low

Abbreviations: N: total number of participants; n: number of participants; EC: clinical trial; RCT: randomized clinical trial; IG: intervention group; CG: control group.

**Table 3:** Interviews conducted in each study.

Study	Interviews Conducted
López et al., [17] 2022.	Presurgical interview the afternoon before the intervention, with the patient on the ward. It consists of a presentation by the nurse herself; detailed explanation of the process, circuit and professionals involved; recommendations for absolute diet, removal of prostheses and other objects; recommendations to relatives about waiting areas, information system and circuit; familiarization with surgical environment; information on pain control systems, including planned rescue medication; presentation of pumps and devices; requests and questions.
Romero et al., [5] 2020.	In addition to the usual procedure, they were given an informative brochure with information on perioperative care. This included: information to relatives; the floor plan and the waiting room and information to patients with the welcome and the steps to follow until discharge.
Abdullah et al., [19] 2015.	Individual briefing on surgical preparation and procedures.
Dias et al., [20] 2022.	Dedicated and standardized personalized preoperative interview conducted by the 10 min certified by nurse, following a patient-centered approach.
Fortacín et al., [21] 2015.	The surgical block nurse provides standardized information along with specific information from the presurgical visit.
Medina et al., [22] 2019.	Nurse carried out an individual and informative session in addition to a motivational interview during a period of 20 days, by a follow-up session 4 weeks later, which was based on the participants establishing by their own goals to gradually change their lifestyle. Each session lasted approximately 40 min, beginning with the exploration of the level of anxiety and triggers during the eight days prior to the interview.

**Table 4:** Leave-one-out sensitivity analysis for preoperative anxiety, postoperative pain, and postoperative anxiety.

Study	Preoperative Anxiety			Postoperative Pain			Postoperative Anxiety		
	MD [95%CI]	*p* Value	I^2^ (%)	SMD [95%CI]	*p* Value	I^2^ (%)	SMD [95%CI]	*p* Value	I^2^ (%)
Abdullah et al., [19] 2015	−0.98 [−4.55, −1.01]	0.08	60	-	-	-	−0.46 [−0.81, −0.10]	0.01	0
Dias et al., [20] 2022	−2.56 [−4.01, −1.11]	0.006	0	−0.81 [−1.03, −0.60]	<0.001	0	−0.55 [−0.93, −0.17]	0.004	0
Medina et al., [22] 2019	−0.98 [−4.55, −2.29]	0.08	60	-	-	-	−0.46 [−0.81, −0.10]	0.01	0
Romero et al., [5] 2020	−0.42 [−4.36, 3.52]	0.16	45	-	-	-	-	-	-
Fortacin et al., [21] 2015	-	-	-	−0.66 [−1.11, −0.22]	0.004	66	-	-	-
Moraleda et al., [17] 2022	-	-	-	−0.50 [−0.85, −0.15]	0.005	0	-	-	-

Abbreviations: MD: mean difference; SMD: standardized mean difference; CI: confidence interval; I^2^: heterogeneity statistic.

## Data Availability

All the databases employed and/or analyzed in this study are available from the corresponding author upon reasonable request.

## Supplementary Materials

The following supporting information can be downloaded at: https://www.mdpi.com/article/10.3390/medicina62061182/s1. PRISMA 2020 Checklist [47].
